# Temporal Petal Closure Benefits Reproductive Development of *Magnolia denudata* (Magnoliaceae) in Early Spring

**DOI:** 10.3389/fpls.2017.00430

**Published:** 2017-03-30

**Authors:** Liya Liu, Chulan Zhang, Xiangyu Ji, Zhixiang Zhang, Ruohan Wang

**Affiliations:** ^1^National Engineering Laboratory for Tree Breeding, Key Laboratory for Genetics and Breeding of Forest Trees and Ornamental Plants, Ministry of Education, College of Biological Sciences and Biotechnology, Beijing Forestry UniversityBeijing, China; ^2^Lab of Systematic Evolution and Biogeography of Woody Plants, College of Nature Conservation, Beijing Forestry UniversityBeijing, China

**Keywords:** adaptation, anther development, floral closure, petal movement, pollination

## Abstract

The Magnoliaceae shows strong phylogenetic niche conservatism, in which temporal petal closure has been extensively reported. However, it is yet elusive whether temporal petal closure is an idle floral character inherited from their ancestors or an adaptive trait to their habitats. Here, we monitored the process of temporal floral closure and re-opening in a thermogenic plant, *Magnolia denudata* (Magnoliaceae). Furthermore, we artificially interrupted temporal petal closure and investigated its effects on development of female and male gametophytes. Intriguingly, we found considerable anatomical changes in the anthers shortly after temporal closure of petals: disintegration of tapeta, crack of anther walls, and release of matured pollens. In comparison with normal flowers, artificially interrupted flowers (no petal closure) showed delayed anther development and slower pollen germination on stigmas, while little difference in embryo morphology was observed during the early stage of embryo development. Moreover, seed set and quality were significantly decreased when petal closure was prevented. In addition, we found pollination accelerated floral closure in *M. denudata*. Taken together, temporal floral closure benefits reproduction of *M. denudata* in early spring by promoting anther development and pollen function, which suggests that it is an adaptive floral trait to its specific habitat.

## Introduction

The flowering stage is of crucial importance to plants since it marks the onset of double fertilization and subsequent seed set, which leads to regeneration of the population. In order to adapt to diverse environments, flowers have evolved wonderful variation in morphology, such as color, shape, and size, which is beneficial for pollinator visiting and protection of internal structures (Clark and Husband, 2007). Except for morphological variations, there are non-morphological changes in flowers, namely floral movement. Many plant species are capable of moving some portions of their anatomy, such as petal and pistil in response to internal and/or external factors (Hase et al., 2006; Ren and Tang, 2012), which could affect reproduction of flowering plants deeply.

As one of the most extensively observed non-morphological changes of flowers, the opening and closure of petals is an important trait of the productive syndrome (van Doorn and Kamdee, 2014). During the anthesis, some flowers maintain open until petal withering, such as rose, while others show temporal closure and repeated opening of petals, such as lotus. From a physiological point of view, flower opening involves a high rate of cell expansion (Singh et al., 2011; Pei et al., 2013). It has attracted extensive attention of scientists by the complex regulation and also has inspired artists and common people by the impressive and emotional petal movements. Although several suspected regulatory mechanisms, such as internal circadian rhythm (Trivellini et al., 2016; Yon et al., 2016), light control (Trivellini et al., 2016), temperature control (Calinger et al., 2013), and moisture control (Magalhaes and Angelocci, 1976), have been raised, ecological roles of the various types of flower opening remain elusive.

*Magnolia denudata* belongs to the Magnoliaceae and usually flowers in cold early spring. In former studies, we found that thermogenic flowers of *M. denudata* were hermaphroditic and protogynous, usually with temporal petal closure occurring during the anthesis (Wang et al., 2013). However, little is known regarding the ecological roles of this temporary petal closure. In this study, we investigated the process of floral opening and closure during the anthesis of *M. denudata*, with emphasis on the effects of temporal floral closure on stamen development, pollen function, embryo development, as well as seed production.

## Materials and Methods

### Study Species and Area

*Magnolia denudata* trees used in this study were located in the campus of Beijing Forestry University (40°00′03″ N, 116°20′25″ E; 68 m a.s.l.), with 10–15 m in height and 20–25 cm in diameter at breast height. The study site had a temperate climate, with mean annual rainfall of 400 mm and mean air temperature of 4–13°C. Trees flower from mid-March to mid-May, with a peak in mid-April.

### Timing of Flower Opening and Closure

Twenty swollen flower buds (nearly open) of *M. denudata* were arbitrarily selected in a sunny morning for observation of flower opening and closure. The outer petals maintained open throughout the anthesis and the inner petals showed repeated opening and closure, thus opening angles of the inner petals were measured to monitor the process of flower opening and closure. Measurements were performed at 2-h intervals from 8:00 to 20:00 each day during the anthesis of each individual flower.

### Effects of Temporal Floral Closure on Stamen Development

**FIGURE 1:**
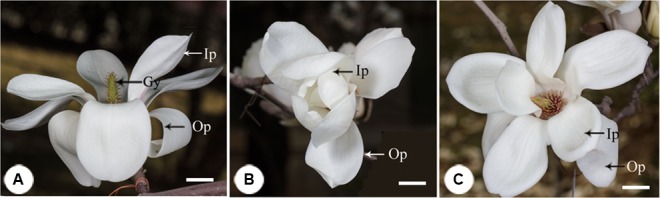
**The process of floral closure and re-opening in *Magnolia denudata*. (A)** Flower was first opening at the pistillate stage. The stigmas were receptive and stamens appressed tightly to the style with no anthers dehiscence. **(B)** Temporal closure of the flower at the post-pistillate stage. The inner petals were closed and formed a chamber in the evening. **(C)** Flowers re-opened at the staminate stage. The gynoeciums withered and the stamens matured with anthers dehisced. Ip, inner petal; Op, outer petal; Gy, gynoecium. Bars: 2 cm.

Based on observation of the timing of flower opening and closure, there were four stages of inner petal movements before floral withered, including pre-pistillate, pistillate, post-pistillate, and staminate stages (see Results, **Figure 1**). Androecia of three flowers were fixed in FAA for each of the four stages. To estimate the effects of temporal floral closure on stamen development, six flowers were prevented from closure by stacking inner petals at the pistillate stage. During the subsequent post-pistillate and staminate stages, androecia were collected and fixed in FAA for these six flowers, with three for each stage. The fixed androecia were dehydrated in an ethylalcohol series, embedded in paraffin wax, sectioned (8–10 μm thickness), and then stained with toluidine blue. All sections were observed under an Olympus BH-2 microscope and photographed using an Olympus DP 70 photo micrography system.

### Effects of Floral Closure on Pollen Function

Six flowers were manually pollinated at the pistillate stage and divided into two groups: three flowers were stuck with gummed tape to prevent floral closure as described above, the rest three were kept at natural conditions and used as a control. Apocarpous pistils were sampled from these flowers to examine pollen germination and pollen tube growth in 6 h after pollination. Apocarpous pistils collection was conducted at a 2-h interval and one flower from each of the stuck and control group was used for each time. The collected apocarpous pistils were fixed in ethanol: acetic acid (3:1) for 30 min and then stored in 70% ethanol until use. After being softened in 1 mol/L NaOH overnight and washed in distilled water for three times, the samples were stained with decolorized aniline blue, squashed, examined, and photographed using a fluorescence microscope (Olympus BX51).

### Effects of Floral Closure on Ovule Development

Twelve flowers were manually pollinated at the pistillate stage and divided into two groups: six flowers were stuck by gummed tape to prevent floral closure as described above, the rest six were kept under natural conditions and used as a control. On the 4th and 8th days, ovules were sampled form three flowers for each of the stuck and control group. The ovule samples were fixed in 2.5% glutaraldehyde and 4% paraformaldehyde for 24 h and sectioned (6 μm thick), stained with toluidine blue, and observed under an Olympus BH-2 microscope. Photographing was performed using an Olympus DP 70 system.

### Effects of Temporal Closure on Seed Production

To estimate the effects of temporary closure on seed production, 20 flowers were manual pollinated in April. Ten of the 20 flowers were prevented from temporary closure as described above, the rest 10 flowers were kept under natural conditions, until seeds produced by these flowers got mature. Fruits from these flowers were collected in September and seed size and seed mass analyzed.

### Effects of Pollination on Floral Closure

Twenty pistilate-stage flowers were chosen in a sunny morning to estimate the effects of pollination on floral closure. Artificial pollination was performed at 10:00 for 10 of the 20 flowers. The rest 10 flowers were covered using plastic meshes to prevent pollination by insects. Movements of the inner petals were examined for the 20 flowers every 2-h before 20:00.

## Results

### Timing of Floral Closure and Re-opening

**FIGURE 2:**
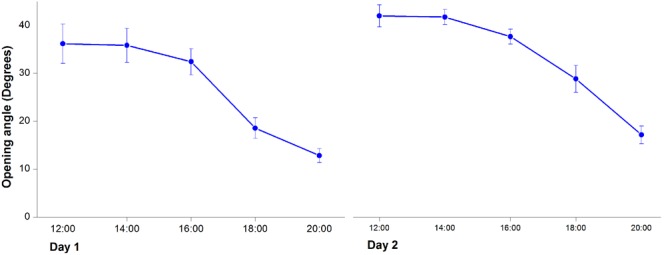
**Changes of opening angles during floral closure and re-opening.** Values are means ± SE.

In the first morning, *M. denudata* flowers were tightly closed at 8:00. The outer petals started to loosen at 10:00, which indicated the onset of flower opening. Then inner petals also opened gradually. The flowers fully opened at 12:00 when the opening angle reached 36.18 ± 0.14° (**Figures 1A, 2**). During this period, stigmas were receptive with some crystalline secretion on the surface and stamens appressed tightly to the style with no anthesis dehiscence, indicative of the pistillate stage of the flowers. The flowers maintained fully-open by 16:00, after which the opening angles decreased sharply. The angles decreased to 12.83 ± 0.08° and the inner petals formed a chamber at 20:00, which marks the temporal closure of the flowers at the post-pistillate stage (**Figures 1B, 2**).

In the second day, inner petals started to open again at 10:00 and fully opened at 12:00 when the opening angles reached 42 ± 0.13° (**Figures 1C, 2**). At this stage, the gynoeciums withered and the stamens matured with anthers dehisced, suggesting that the flowers entered the staminate stage. Inner petals of the fully opened flowers started to close again after 16:00 and formed chambers with the inner petals at 20:00 as they did in the first day. In the third morning, the inner petals re-opened again at 10:00 and maintained open until the flowers withered.

### Effects of Temporal Floral Closure on Male and Female Gametophytes

**FIGURE 3:**
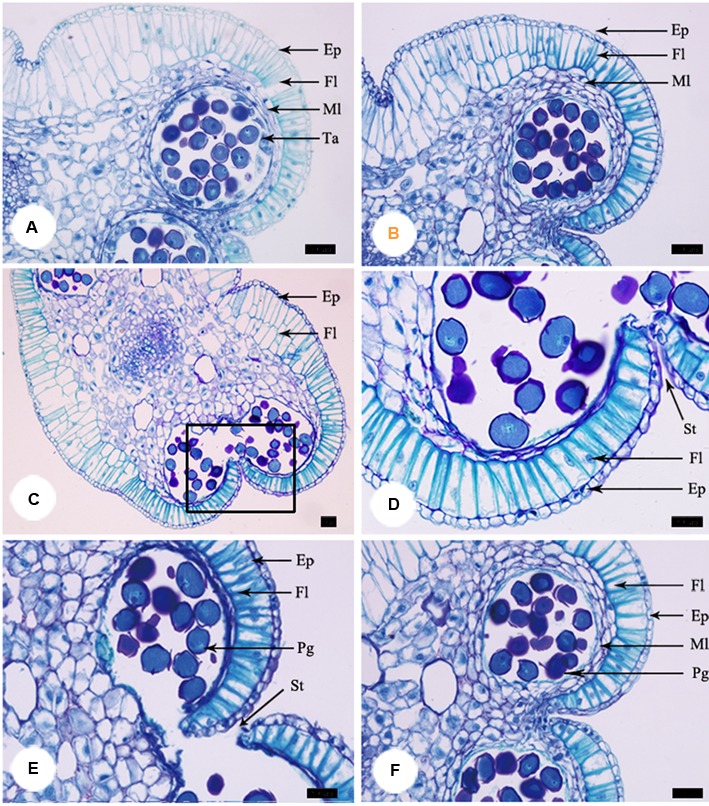
**Anther development during the process of floral closure and re-opening. (A)** Anther was intact and no pollen was released at the pre-pistillate stage. **(B)** Tapetum began to disintegrate gradually at the pistillate stage. **(C)** The tapetum disappeared and stomium formed at the post-pistillate stage. **(D)** A higher magnification of the region of interest in **(C)**. **(E)** Stomium ruptured, fiber layers expanded, connective cells broke down, and pollen grains released at the staminate stage. **(F)** Anther of stuck flowers at the staminate stage. Ep, epidermis; Fl, fiber layer; Ml, middle layer; Ta, tapetum; St, stomium. Bars: 50 μm.

At the pre-pistillate stage, anthers stayed intact and no pollen was released. The tetrasporangiate anther wall consisted of epidermis, endothecium, 2–3 middle layers, and glandular tapetum of 1–2 cells (**Figure 3A**). When the inner petals were opened for the first time and flowers entered the pistillate stage, tapetum began to disintegrate gradually (**Figure 3B**). The tapetum totally disappeared and stomium formed at the junction of two pollen sacs at the post-pistillate stage (**Figures 3C,D**). In the second morning, inner petals re-opened and flowers were at the staminate stage. The stomium ruptured, the fiber layers expanded, and the connective cells broke down, which led to release of pollen grains (**Figure 3E**). When the inner petals were stuck to disrupt floral closure at the pistillate stage, anther dehiscence and pollen release were observed 18 h later than the non-stuck control flowers (**Figure 3F**).

**FIGURE 4:**
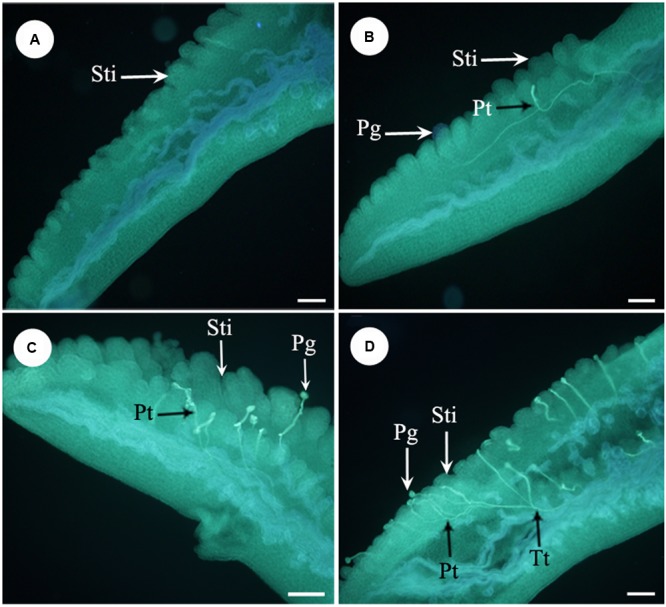
**Fluorescence micrographs of pollens in the apocarpous gynaecium. (A)** No pollen germinated on stigmas of stuck flowers 4 h after artificial pollination. **(B)** Pollens started to germinate 4 h after artificial pollination of non-stuck flower. **(C)** Pollens start to germinate 6 h after pollination in stuck flowers. **(D)** Pollen tubes entered the transmitting tissue in non-stuck flowers 6 h after pollination. Sti, stigma; Pg, pollen grain; Pt, pollen tube; Tt, transmitting tissue. Bars: 100 μm.

**FIGURE 5:**
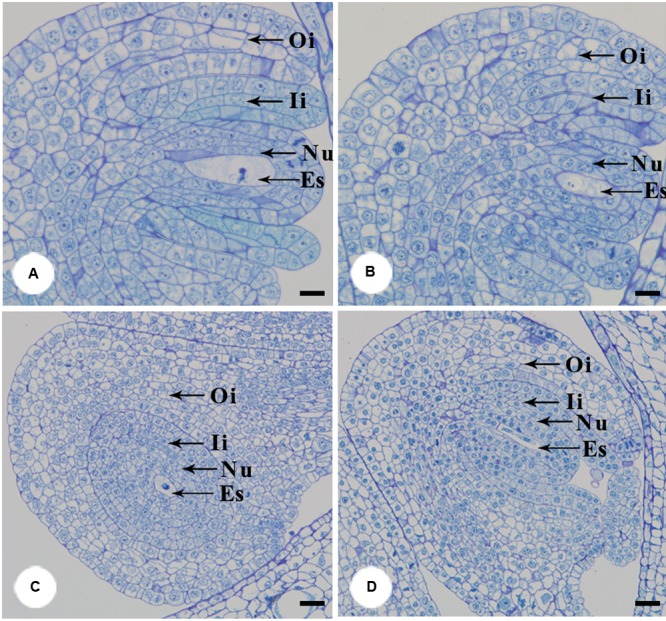
**Anatomical structures of ovule after artificial pollination.** The functional megasporeis, inner and outer integuments developed of stuck **(A)** and non-stuck flowers **(B)** on the 4th day after pollination. Growth of inner and outer integument of stuck **(C)** and non-stuck **(D)** flowers on the 8th day after pollination. Ii, inner integuments; Oi, outer integuments; Nu, nucellus; Es, embryo sac. Bars: 20 μm.

Furthermore, we examined pollen germination and pollen tube growth after artificial pollination. In non-stuck flowers, pollen germination was observed 4 h after pollination (**Figures 4A,B**). In stuck flowers, pollens did not start to germinate until 6 h after pollination, by which time some pollen tubes entered the transmitting tissue in the styles of non-stuck flowers (**Figures 4C,D**). The embryo sacs of *M. denudata* were immature at the pistillate stage. When the petals were stuck at the pistillate stage after artificial pollination, the embryo development showed little difference from that of non-stuck flowers in 8 days (**Figure 5**).

### Effects of Temporal Floral Closure on Seed Set

**FIGURE 6:**
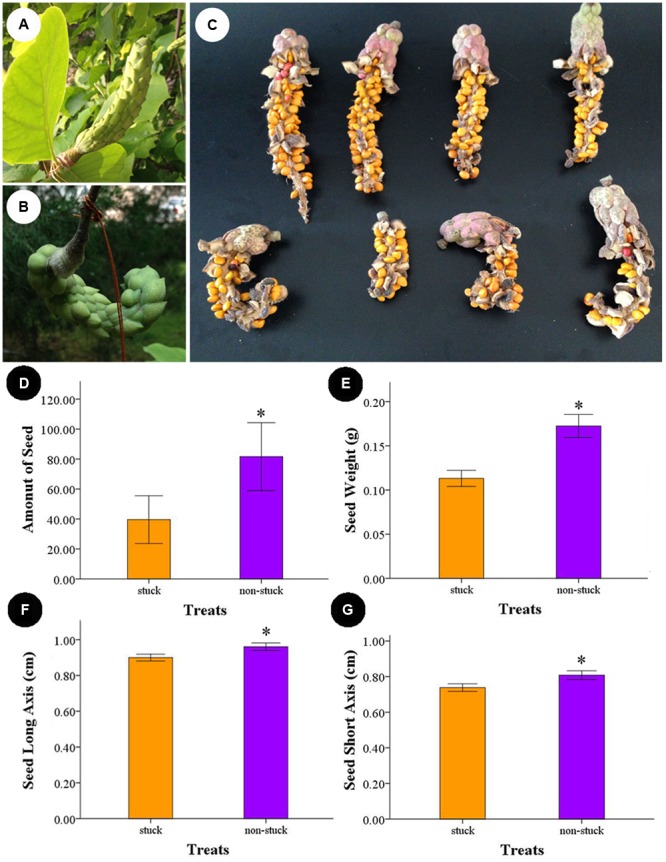
**Follicle and seeds produced by non-stuck and stuck flowers. (A)** Young fruits produced by non-stuck flowers were plump and erect. **(B)** Young fruits produced by stuck flowers were curled. **(C)** Seeds in the follicle produced by non-stuck and stuck flowers. Comparison of seed production between non-stuck and stuck flowers: seed set **(D)**, seed weight **(E)**, and long **(F)** and short **(G)** axis of seeds. Asterisks indicate significant difference (*p* < 0.05) between non-stuck and stuck flowers.

The carpals began to expand 10 days after artificial pollination. The follicles produced by non-stuck flowers were erect and grew to as long as 18 cm. For the stuck flowers, follicles were wound in shape (**Figure 6**). Follicles contained an average of 81.55 ± 9.82 seeds for the non-stuck flowers (*n* = 9, for one fruit was lost before ripening). The stuck flowers produced 39.60 ± 7.03 seeds per follicle, which was significantly (*p* < 0.05) fewer than that of non-stuck flowers (**Figure 6**). Seed mass was 0.17 ± 0.03 g per seed (*n* = 30) for the non-stuck flowers, which was significantly higher than that of stuck flowers (0.11 ± 0.02 g per seed, **Figure 6**). Seeds size was also significantly smaller for stuck flowers than non-stuck flowers (**Figure 6**).

### Effects of Pollination on Floral Closure

When the pistillate-stage flowers were artificially pollinated, inner petals began to close at16: 00. After 2 h, 9 out of the 10 flowers were fully closed, and the rest one was nearly closed. For the non-pollinated flowers, closure of inner petals was also observed whilst the closure was much slower. When the inner petals of pollinated flowers fully closed, only 2 out of 10 were nearly closed for the non-pollinated flowers and the rest eight flowers were in the process of closure, with an average opening angle of 33.8 ± 2.6°.

## Discussion

The flowering stage, in which pollination and fertilization occur, leading to seed set in female and bisexual flowers, is a crucial period for plant life history. There is great variation of floral period among different plants; it can range from a few minutes to days or even months (Ashman and Schoen, 1994; Clark and Husband, 2007). Some flowers remain open and functional during the anthesis, while others could show repeated opening and closure (Abdusalam and Tan, 2014). In the present study, *M. denudata* flowers showed a repeated and diurnal opening in response to the day/night cycle during its anthesis. It is intriguing to note that there is vast variation of flower opening types within the *Magnolia* genus. *Magnolia sprengeri*, like *M. denudata* in the current study, flowers in daytime (Wang et al., 2014), while *M. ovata* and *M. virginiana* in night (Gottsberger et al., 2012; Losada et al., 2014). Some *Magnolia* species, such as *M. praecocissima, M. schiedeana*, and *M. tamaulipana* have a single flower opening (Dieringer and Espinosa, 1994; Ishida, 1996; Dieringer et al., 1999), while others show repetitive opening, such as *M. denudata, M. sprengeri, M. ovata*, and *M. virginiana* (Wang et al., 2010, 2014; Gottsberger et al., 2012; Losada et al., 2014). Considering that these *Magnolia* species are living in different habitats and pollination was mediated by different insects (Dieringer et al., 1999; Gottsberger et al., 2012; Wang et al., 2014), the vast variance in flowering types might be an adaptation to their pollinators with distinct activity rhythms.

A “chamber” was formed when the flowers of *M. denudata* temporally closed, which has also been frequently reported in some other thermogenic plants (Seymour et al., 2009a, 2010; Dieringer et al., 2014). There are different ways to form a floral chamber for thermogenic plants. For *Arum* species, the spathes directly develop into a chamber shape (Seymour et al., 2009a). Floral chambers are formed as a result of partial open of the petals at the female stage in *M. ovata* (Seymour et al., 2010). Being different from *Arum* species and *M. ovata, Nelumbo lutea* flowers form chambers by temporal closure of the petals (Dieringer et al., 2014). In *M. denudata*, we found similar situation to *N. lutea*, with a slight difference that the temporal closure of the flowers was restricted to inner petals.

Despite of different ways to form floral chambers, it has been putatively assumed that floral chambers can attract pollinators to stay in the flowers for longer time by providing a favorable micro-environment for foraging and mating (Seymour et al., 2010; Gottsberger et al., 2012; Dieringer et al., 2014). In this study, pollen dehiscence was delayed, pollen germination was low, and pollen tube growth was slow, when the floral chamber was disturbed in *M. denudata*. It has been demonstrated in plants with both thermogenic and non-thermogenic flowers that pollen function was considerably affected by temperature (Seymour et al., 2009b; Coast et al., 2016), which might involve regulation mediated by the GA pathway and Ca^2+^ signals (Mähs et al., 2013; Sakata et al., 2014). Thus, retardance of heat loss by the floral chamber may also play a role in facilitating pollen function. Although little anatomical difference was observed in the embryo at the early stage between stuck and non-stuck flowers, seed set and seed mass were significantly decreased when petal closure was disturbed for *M. denudata*, suggesting the importance of floral chamber in seed development at the post-embryonic stage. Our findings suggested new ecological roles of floral chambers other than attracting pollinators by heat reward.

Floral open and closure involve complex regulatory mechanisms. Since the concept of “floral clock” was proposed by Linné (1783), the circadian pattern of floral opening and closure has been more and more widely appreciated (Burghardt et al., 2016; Mora-García et al., 2017). Besides the endogenous circadian rhythm of flowers, daily changes of light/dark and temperature were also regarded to take roles in regulation of the circadian rhythm (Johansson and Staiger, 2015; Burghardt et al., 2016). Here, we found that pollinated flowers closed considerably earlier than non-pollinated flowers in *M. denudata*. For single opening flowers, an earlier closure may save some energy for the plants. The ecological advantages of early closure after pollinator remain unclear for repeated opening flowers. Some Asteraceae flowers were also reported to show earlier closure after pollination (Fründ et al., 2011). These results provided new clues for investigating the regulatory framework of floral opening and closure.

In summary, *M. denudata* showed repeated closure of inner petals in night to form floral chambers. We demonstrated new ecological roles of floral chambers in facilitating pollen function and seed development, besides the commonly suspected role of favorable micro-environment for pollinators. In addition, the vast variance in types of floral opening and closure within the *Magnolia* genus may provide diverse genetic resources for studying phylogeny and ecological roles of temporal floral closure.

## Author Contributions

Conceived and designed the experiments: RW, LL, CZ. Performed the experiments: LL, CZ, XJ. Analyzed the data: LL, CZ, RW. Wrote the paper: RW. Provided crucial suggestion on the experiment: ZZ.

## Conflict of Interest Statement

The authors declare that the research was conducted in the absence of any commercial or financial relationships that could be construed as a potential conflict of interest.
